# Left Vocal Cord Paralysis Detected by PET/CT in a Case of Lung Cancer

**DOI:** 10.1155/2015/617294

**Published:** 2015-11-03

**Authors:** Ali Ozan Oner, Adil Boz, Evrim Surer Budak, Gulnihal Hale Kaplan Kurt

**Affiliations:** ^1^Nuclear Medicine Department, School of Medicine, Afyon Kocatepe University, 03200 Afyon, Turkey; ^2^Nuclear Medicine Department, School of Medicine, Akdeniz University, 07070 Antalya, Turkey; ^3^Nuclear Medicine Department, Antalya Training and Research Hospital, Antalya, Turkey; ^4^Nuclear Medicine Department, Isparta State Hospital, Isparta, Turkey

## Abstract

We report a patient with lung cancer. The first PET/CT imaging revealed hypermetabolic mass in the left aortopulmonary region and hypermetabolic nodule in the anterior segment of the upper lobe of the left lung. After completing chemotherapy and radiotherapy against the primary mass in the left lung, the patient underwent a second PET/CT examination for evaluation of treatment response. This test demonstrated, compared with the first PET/CT, an increase in the size and metabolic activity of the primary mass in the left lung in addition to multiple, pathologic-sized, hypermetabolic metastatic lymph nodes as well as multiple metastatic sclerotic areas in bones. These findings were interpreted as progressive disease. In addition, an asymmetrical FDG uptake was noticed at the level of right vocal cord. During follow-up, a laryngoscopy was performed, which demonstrated left vocal cord paralysis with no apparent mass. Thus, we attributed the paralytic appearance of the left vocal cord to infiltration of the left recurrent laryngeal nerve by the primary mass located in the apical region of the left lung. In conclusion, the knowledge of this pitfall is important to avoid false-positive PET results.

## 1. Introduction

Vocal cord paralysis occurs due to pathologies of the nerves that innervate vocal cords. The nerves that make vocal cords vibrate consist of neurons originating from the region of nucleus ambiguus in the brainstem. The nerve arising from the nucleus is called the “vagus nerve,” which is the thickest nerve in the human body and extends to thoracic and abdominal cavities [1]. The nerve gives off 2 thin branches for larynx at the base of the skull. The first one is called the “superior laryngeal nerve” and the second one is called the “recurrent laryngeal nerve (RLN).” The latter conveys orders to both opening and closure muscles. The problems in that nerve cause paralysis of both opening and closure muscles, leading to loss of their basic functions. Hence, respiratory difficulty and hoarseness and aspiration problems due to failure of closure arise. Problems of superior laryngeal nerve, on the other hand, become manifest, with a monotonous, thin voice as well as difficulty in tone control and singing songs.

Causes of Vocal Cord Paralysis:

- Idiopathic diseases.
- Viral neuritis.
- Masses, tumors compressing vocal nerves in brain, base of the skull, neck, thyroid region, and thoracic cavity.
- Surgical interventions (especially thyroid surgery).
- Being secondary to intubation in certain surgical operations [1].

^18^F-fluorodeoxyglucose positron emission tomography/computed tomography (^18^FDG-PET/CT) scans are utilized for identification of stage cancers clinically but the causes of false-positive and false-negative results must be identified to evaluate the results of the test. As a result of the increased glucose consumption, FDG accumulates in benign and malignant conditions. The degree of muscle work is directly commensurate with the amount of glucose being dealt with [2].

The lack of FDG activity in the paralyzed cord and compensatory activation of the nonparalyzed vocal cord causes asymmetric FDG uptake seen in vocal cord paralysis. In vocal cord paralysis, workload of the nonparalyzed cord increases and so glucose consumption increases which is seen as a focal hotspot on FDG PET images [3].

We report a case of lung cancer in a patient with a false-positive PET scan in the larynx due to increased workload of the right vocal cord as it compensates for the paralyzed left vocal cord.

## 2. Case History 

**Figure 1:**
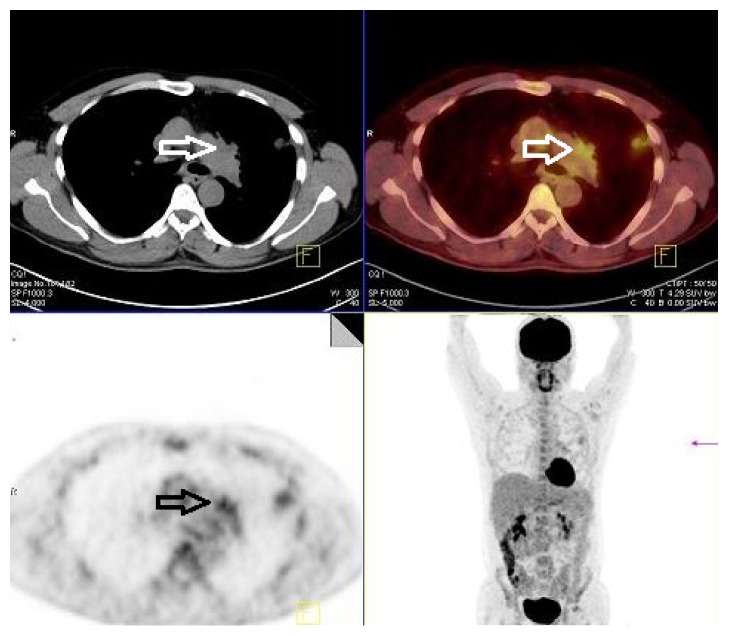
First PET/CT: axial CT, pet, fused PET/CT, and coronal MIP images revealed a hypermetabolic mass (arrows) in the left aortopulmonary region and hypermetabolic nodule in the anterior segment of the upper lobe of the left lung.

A 46-year-old man presented to Faculty of Medicine, Akdeniz University, with dyspnea, cough, and sputum. He was diagnosed with small cell lung cancer as a result of examination of a left lung biopsy. The first PET/CT imaging dating revealed hypermetabolic mass in the left aortopulmonary region and hypermetabolic nodule in the anterior segment of the upper lobe of the left lung (Figure 1).

**Figure 2:**
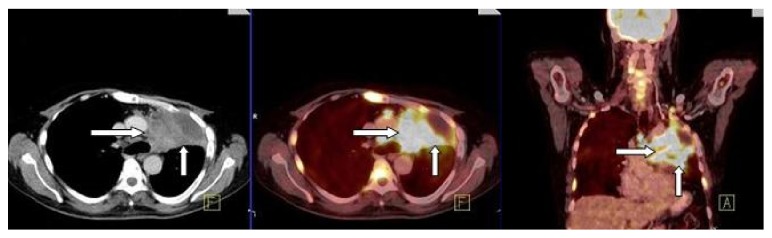
Second PET/CT: axial CT, axial PET/CT, and coronal PET/CT images revealed an increase in the size and metabolic activity of the primary mass in the left lung and mediastinal lymph nodes (arrows) when we compared with first PET/CT images.

After completing chemotherapy and radiotherapy against the primary mass in the left lung, the patient underwent a second PET/CT examination for evaluation of treatment response. It revealed, compared with the first PET/CT, an increase in the size and metabolic activity of the primary mass in the left lung, multiple, pathologic-sized, hypermetabolic metastatic lymph nodes (Figure 2), and multiple metastatic sclerotic areas in bones, consistent with progression of disease.

**Figure 3:**
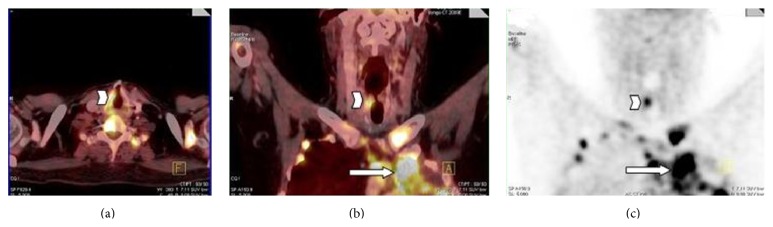
Second PET/CT: (a) transverse, (b) coronal PET/CT fusion, and (c) coronal PET images show intense FDG uptake in the left lung tumor (arrow) and in the right vocal cord (arrowhead).

In addition, an asymmetrical FDG uptake was noticed at the level of right vocal cord (Figure 3). During follow-up, the patient was sent for laryngoscopy, which demonstrated left vocal cord paralysis with no apparent mass. Thus, we considered that the paralytic appearance of the left vocal cord may have been due to infiltration of the left recurrent laryngeal nerve by the primary mass observed in the apical region of the left lung. The asymmetrical FDG uptake in the right vocal cord was attributed to overactivity of right vocal cord muscles to compensate left vocal cord paralysis.

## 3. Discussion

In the course of PET/CT scans, the patient must rest in silence, without any movement during the uptake period of FDG. After injection of isotope, if the patient coughs, talks, or chews, we can see a normal glucose uptake by larynx, tongue, and pharyngeal musculature. Generally this uptake is symmetric and can be intense, moderate, or mild [4].

The suspicion of a primary neoplastic or inflammatory cord pathology should be always enhanced by asymmetric increased FDG uptake. In identifying primary and recurrent laryngeal cancer, FDG PET has been found to be useful [5, 6]. Asymmetric increased uptake can be observed due to impaired movement or paralysis of the contralateral vocal cord [7–11]. When there is incidental detection of asymmetric vocal cord activity, the clinical history often helps us. An evolution of prior surgery, hoarseness, or intervention in the thyroid, larynx, neck, or mediastinum are marks to indicate injury of one of the recurrent laryngeal nerves. Laryngoscopic examination will help us to see impaired movement or paralysis of the contralateral vocal cord and, at the same time, we can also see if there is a primary pathology in the ipsilateral cord [3].

Symmetric vocal cord uptake is the possibility of physiologic, either in patients with normal resting cords or in patients vocalizing at or soon after FDG injection. If there is asymmetric FDG uptake in vocal cord activity in an oncology patient, we should suspect for RLN palsy. Pathologies being found throughout the course of the recurrent laryngeal nerves, such as enlarged lymph nodes or masses in the root of the neck or the mediastinum, can infiltrate the nerves which trigger vocal cord paralysis.

Vocal cord paralysis is more common due to left recurrent laryngeal nerve infiltration than right recurrent laryngeal nerve infiltration. This is because the left recurrent laryngeal nerve has a longer anatomical pathway and passes through the aortopulmonary window. Kamel et al., in a study on 184 patients with lung cancer, reported 6 cases of vocal cord paralysis, all of which were due to infiltration of left recurrent laryngeal nerve [11].

However, there are also cases of vocal cord paralysis due to right recurrent laryngeal nerve paralysis in the literature. Purandare et al. reported a hypermetabolic metastatic lymph node of pathologic size at level 4 in neck in a patient with esophagus cancer. They reported that that lymph node infiltrated and paralyzed right recurrent laryngeal nerve and led to asymmetrical FDG uptake [3].

Minamimoto et al., in a study in 59 patients with vocal cord paralysis, observed that the asymmetrical FDG uptake was on the ipsilateral side with the lesion when the primary lesion was in laryngeal region whereas on the contralateral side with the lesion when the lesion infiltrated recurrent laryngeal nerve. Both conditions in that study were characterized by a significant difference in maximum standardized uptake values (SUVmax) of FDG uptake in vocal cords [12].

## 4. Conclusion

In this case, PET/CT images demonstrated that the focal FDG uptake was localized in the right vocal cord muscles. This focal FDG uptake was a result of increased workload of vocal cord muscles caused by contralateral RLN palsy due to direct nerve invasion by lung cancer of the left lung apices. The knowledge of this pitfall is important to avoid false-positive PET results.
